# POSTING SHORT-FORM VIDEOS PROMOTES OLDER ADULTS’ PSYCHOLOGICAL HEALTH: A MODEL BASED ON SELF-DETERMINATION THEORY

**DOI:** 10.1093/geroni/igae098.3539

**Published:** 2024-12-31

**Authors:** Jingxuan Wu, Huamao Peng

**Affiliations:** Beijing Normal University, Beijing, Beijing, China (People’s Republic); Beijing Normal University, Beijing, Beijing, China (People’s Republic)

## Abstract

**Objectives:**

This study focuses on how posting behaviors of older adults on short-form video platforms (e.g., TikTok) influences depression and reveals the psychological mechanism (satisfying basic psychological needs of autonomy, competence, and relatedness) from the perspective of the Self-Determination Theory and uncover whether browsing behavior moderates this effect. Method: A total of 1705 older adults aged 55 to 83 years were instructed to complete the Center for Epidemiologic Studies Depression Scale, the Short-form Video Usage Behavior Questionnaire, and the Basic Psychological Needs Scale. Data analysis was conducted using SPSS 25.0.

**Results:**

1) Older adults posting short-form videos exhibit lower levels of depression compared to those who have never posted short-form videos through the mediations of greater competence (indirect effect = -.15) and autonomy (indirect effect = -.05), and the indirect effect of autonomy becomes insignificant when the diversity of browsing behaviors is high. 2) Among older adults posting short-form videos, posting short-form videos frequently decreases depression through competence (indirect effect = -.16), autonomy (indirect effect = -.04), and relatedness (indirect effect = -.06), regardless of the diversity of browsing behaviors.

**Discussion:**

This study highlights the positive effect of posting short-form videos on older adults’ psychological health by satisfying basic psychological needs. For those unable or unwilling to post videos, diversifying browsing behaviors can improve autonomy and safeguard psychological health. These findings emphasize older adults’ initiative and creativity, along with the importance of lifelong competence development, and present new strategies to support successful aging in the era of Internet-based media.

